# Dynamics on Networks: The Role of Local Dynamics and Global Networks on the Emergence of Hypersynchronous Neural Activity

**DOI:** 10.1371/journal.pcbi.1003947

**Published:** 2014-11-13

**Authors:** Helmut Schmidt, George Petkov, Mark P. Richardson, John R. Terry

**Affiliations:** 1 College of Engineering, Mathematics and Physical Sciences, University of Exeter, Exeter, United Kingdom; 2 Institute of Psychiatry, Kings College London, London, United Kingdom; Brain and Spine Institute (ICM), France

## Abstract

Graph theory has evolved into a useful tool for studying complex brain networks inferred from a variety of measures of neural activity, including fMRI, DTI, MEG and EEG. In the study of neurological disorders, recent work has discovered differences in the structure of graphs inferred from patient and control cohorts. However, most of these studies pursue a purely observational approach; identifying correlations between properties of graphs and the cohort which they describe, without consideration of the underlying mechanisms. To move beyond this necessitates the development of computational modeling approaches to appropriately interpret network interactions and the alterations in brain dynamics they permit, which in the field of complexity sciences is known as dynamics on networks. In this study we describe the development and application of this framework using modular networks of Kuramoto oscillators. We use this framework to understand functional networks inferred from resting state EEG recordings of a cohort of 35 adults with heterogeneous idiopathic generalized epilepsies and 40 healthy adult controls. Taking emergent synchrony across the global network as a proxy for seizures, our study finds that the critical strength of coupling required to synchronize the global network is significantly decreased for the epilepsy cohort for functional networks inferred from both theta (3–6 Hz) and low-alpha (6–9 Hz) bands. We further identify left frontal regions as a potential driver of seizure activity within these networks. We also explore the ability of our method to identify individuals with epilepsy, observing up to 80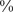 predictive power through use of receiver operating characteristic analysis. Collectively these findings demonstrate that a computer model based analysis of routine clinical EEG provides significant additional information beyond standard clinical interpretation, which should ultimately enable a more appropriate mechanistic stratification of people with epilepsy leading to improved diagnostics and therapeutics.

## Author Summary

In this paper we show that within modular networks (that is, networks with multiple scales of connections), two distinct mechanisms may drive the emergence of synchrony at the global level. We term the first of these mechanisms “network-driven synchrony”, which is characterized by the presence of cycles within the macroscopic network. The second mechanism we term “node-driven”, which is characterized by the ability of an individual node (or nodes) to drive synchrony across the rest of the network, due to the hierarchical structure of the macroscopic network. By applying this framework to routine clinically collected resting state data from people with idiopathic generalized epilepsy and from age matched healthy controls, we demonstrate that functional networks of people with epilepsy have a significantly enhanced capacity to synchronize than those of people without epilepsy. This finding suggests a critical role for the connectivity structure of large-scale networks in the tendency to have seizures. Further, by deriving a mathematical equation for the global synchrony of the network, we make it computationally tractable to analyze data in close to real time. This gives our method potential to be used within the clinic as a diagnostic aid for clinicians treating neurological disease.

## Introduction

The human brain is perhaps the best example of a multiscale complex network, with organizational hierarchies spanning many spatial and temporal scales. At the microscale, neurons communicate with other neurons through both chemical and electrical coupling, with estimates varying from 1000 to 10000 synapses per individual neuron [1]. At the mesoscale within the cerebral cortex, networks of between fifty and one hundred and fifty neurons form minicolumns, which in turn aggregate to form cortical columns, each containing around 5000 neurons [2]. At the macroscale, networks of tightly coupled cortical columns form distinct regions of the cerebral cortex that communicate with each other in a functionally specific manner. There is now increasing evidence for the concept of a core of such brain regions that form structural hubs that are essential for facilitating normal cognitive processing [3]–[5]. Whilst the precise mechanisms by which communication between large-scale brain regions occurs remains an open question, it is widely accepted that many critical brain functions such as cognition and motor coordination result from the emergent dynamics of large networks of neurons [6] and phase synchronization across regions is thought to play a critical role in facilitating communication between regions [7], [8].

From an experimental perspective, a window into the underlying macroscopic structural network may be given by functional networks that can be inferred from imaging modalities such as fMRI, MEG or EEG [9], and a substantial number of methods has been developed and applied to derive functional networks, ranging from cross-correlation [10] and phase coherence [11], to Granger causality [12] and transfer entropy [13], [14]. Whilst strong functional connectivity during the resting state has been shown to be a good indicator of underlying structural connectivity [15], [16], it is important to note that there is no one-to-one translation, thus a degree of fluctuation in functional connectivity is to be expected.

From a theoretical perspective, effective connectivity can be considered a mathematical model driven representation of the functional connectivity inferred from data space [17], and to understand differences between cohorts of patients and controls, several recent studies have used methods from the mathematical field of graph theory [9] to explore either effective or functional networks across a variety of neurological conditions including schizophrenia, dementia and Parkinson's [18]–[21]. A further debilitating neurological disorder that is associated with abnormal synchronization between brain regions is epilepsy; a disorder characterized by the tendency to have recurrent seizures. The International League Against Epilepsy (ILAE) define an epileptic seizure to be “a transient occurrence of signs and/or symptoms due to abnormal excessive or synchronous neuronal activity in the brain” [22]. The role of neural synchronization in seizures has caused some controversy in recent years, in part due to how synchrony is defined [23]. For example, if synchrony at the microscale is strictly defined as single-unit action potentials occurring at the same instance in time, then it can appear that synchrony is decreased during seizures [24]. However, if a broader definition of synchrony, such as phase coherence or generalized synchronization [25], [26], is applied to macroscopic recordings such as EEG then evidence for hypersynchronous activity is commonplace [27]–[30].

**Figure 1:**
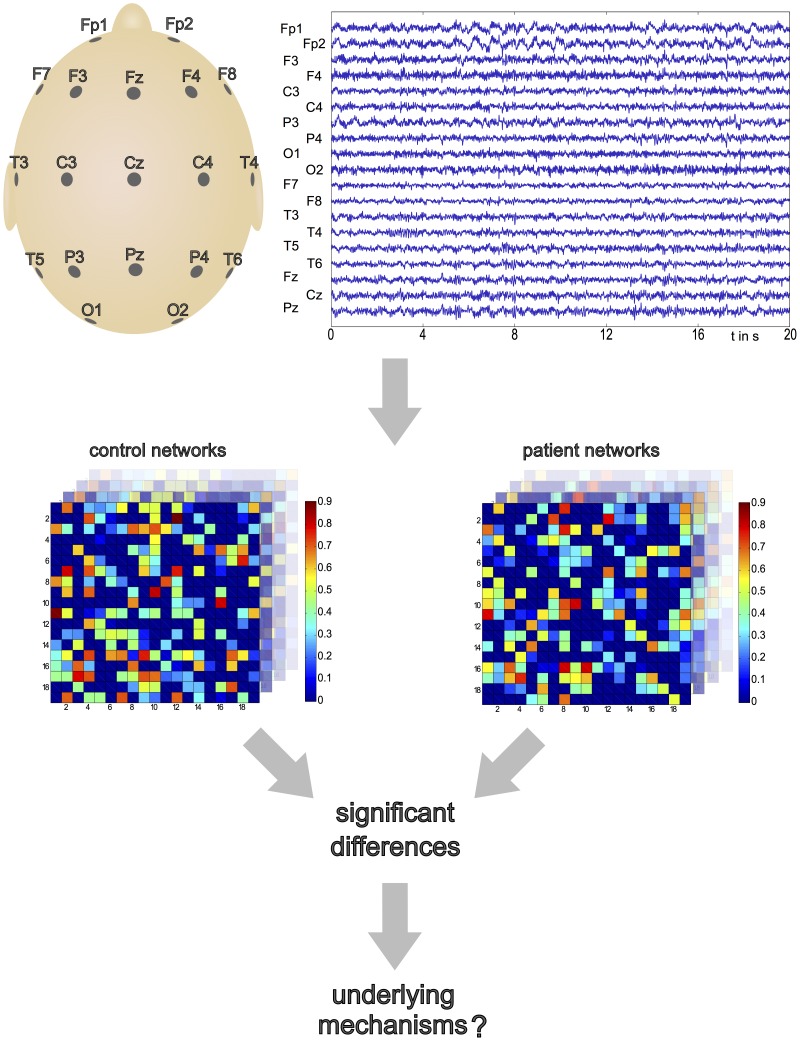
Motivation of our modeling approach. The Electroencephalogram (EEG) records electrical signals from electrodes placed on the scalp. There exist various methods to derive functional network structure from the recorded time series. The primary challenge is to identify (statistically) significant differences between the functional networks of subjects with a particular neurological disorder, and healthy controls. The second challenge is to identify the underlying mechanisms that lead to these changes in network structure, and how they affect the behavior of the model constituents, i.e. the different brain regions. The EEG epochs used in this study are chosen from resting-state, eyes closed. For those subjects with epilepsy, epochs have been selected by a clinically trained expert and are far away from seizures.

An open-question when pursuing a purely graph-theoretic approach is the relationship between the observed network structure and the emergent dynamics supported by that structure; particularly if alterations in function relate to symptoms of the neurological disease (Figure 1). To address this question, it is necessary to introduce a model of the dynamics of each node within the network, and to study the interplay between local dynamics and network structure on the emergent activity. Mathematically, a number of approaches has been used to study the mechanisms of seizure activity. At the physiological level, the use of neural mass and neural field models [31], [32] has become increasingly established to describe the evolution of both spike-wave discharges [33]–[35] and focal epilepsies [36], [37]. These frameworks have enabled important steps toward patient specific representations of these models to be taken using both genetic algorithms [38] and Kalman filtering [39]. Alternatively, at the opposing level of detail, phenomenological models are used to qualitatively describe the critical features associated with different brain states [40]–[43]. These models are typically computationally inexpensive (at least for small networks) making them potentially applicable in a clinical setting, however, they are often only suitable for considering a network at a single level of description and thus represent a coarse simplification of the underlying neurobiology.

In this paper we pursue this phenomenological approach, but choose a model – the Kuramoto model [44] – that is more naturally suited to elucidate the mechanisms by which multiscale network structures can lead to hypersynchrony within or between large-scale brain regions. The Kuramoto model has become a standard model to study synchronization phenomena across physics, chemistry, biology and neuroscience (see [45]–[47] and references therein). Mathematically, the relationship between the Kuramoto model and the Wilson-Cowan model [48], which is a prototypical neural mass model, has been established by Schuster and Wagner [49], [50], and more recently by Daffertshofer and van Wijk [51]. This transition is made in the limit of weak coupling, and as a consequence the amplitude of the original model is neglected. For our purpose, we treat the Kuramoto model as a purely phenomenological model (we show that it mimics the critical features of both background activity and seizures), enabling us to analytically study synchronization phenomena in large-scale networks. As a result we are not limited to the case of weak coupling, since we are not attempting to relate back to a more detailed physiological model.

**Figure 2:**
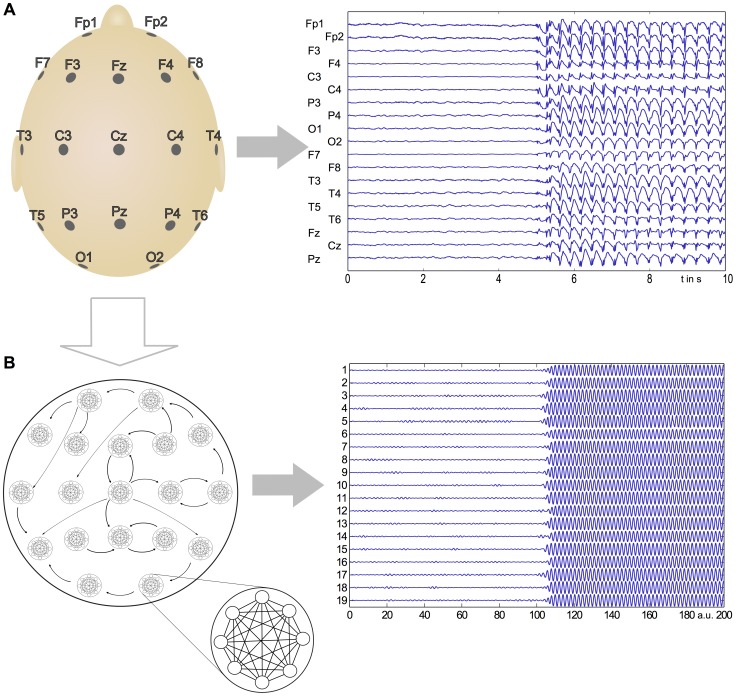
Comparison of recorded seizure with model output. Here we present a comparison between the clinically recorded onset of a generalized seizure event and the output of the modular Kuramoto network, demonstrating that critical features of this transition are captured by the phenomenological model. **A**: Epileptic seizure as captured by EEG. **B**: The Kuramoto model displays behavior similar to epileptic seizures. In the model, we assume local networks (where a node represents a recording site) to be all-to-all connected, analogous to a collection of cortical columns. These nodes are then directionally connected, resulting in a modular network with two scales of coupling.

Instead, the approach we pursue is to consider brain activity, for example as reflected in the macroscopic electrical activity measured using EEG, to be the result of networks of oscillators coupled at two distinct scales of activity: The macroscale electrical activity that is recorded by a scalp electrode is the sum over the dipoles generated by cortical columns (mesoscale) in the vicinity of the electrode. We assume these cortical columns to be strongly connected at close range and form a fully connected network (a so-called complete graph) (Figure 2). In turn, each of these connected networks forms a node (or module) within a larger network, the structure of which is defined by positions of the scalp electrodes. At this larger scale, separate brain regions may share connections, yet do not form complete graphs. Instead the network structure is typically sparse and directed.

The remainder of our paper is arranged as follows. First we introduce the mathematical framework, the method we use to derive functional networks from EEG data, and details of the statistical analysis we perform. Next, we present our results in three parts. In the first part, we demonstrate the conditions required for the emergence of global synchrony across a network. In the second part, we use simple motifs to illustrate how subtle changes in the connectivity structures at different scales can have a dramatic influence on the degree of emergent synchronization, and further illustrate particular structures that can support the emergence of synchrony across either part of or the whole motif. In the final part of our results, we infer networks using clinically recorded EEG from a cohort of people with heterogeneous idiopathic generalized epilepsies, as well as a cohort of healthy controls. We then use our mathematical framework to explore the mechanisms by which seizures can emerge from these networks and find statistically significant differences in the networks of our epilepsy cohort, further demonstrating their potential predictive value at the individual level. We conclude with a discussion of the significance of our findings from both a theoretical and clinical perspective, some limitations of our approach, and suggest avenues for future research.

## Materials and Methods

### Mathematical models

We build a modular network of 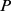 nodes, using the Kuramoto model as a basis for each node 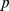:
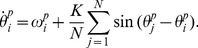
(1)The Kuramoto model is a mathematical representation of a network of 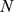 oscillators, coupled together uniformly with strength 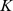 through their phase 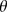. 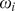 is the natural frequency of the 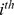 oscillator, and we assume all these frequencies are drawn from a normal distribution function with mean 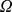 and standard deviation 1. This model has previously been used to study neural oscillations, for example dynamic connectivity mimicking synaptic plasticity [46], [52], planar models with specific synaptic footprint [47], as well as the study of large-scale neural activity on realistic structural networks [53]. Here we consider each oscillator to represent the activity of a mass of neurons, such as a cortical column, for example.

We couple together 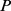 such nodes, each consisting of 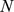 oscillators (in contrast to previous studies, which use one node per network, such as [51] or [54]), following the approach of Barreto *et al*. [55]. Here we introduce a 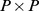 coupling matrix 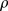 with entries 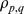 to describe the interaction between nodes 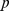 and 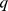, weighted by a global coupling parameter 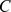:
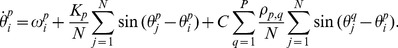
(2)The global connectivity matrix 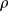 may be either a binary or weighted network, and for mathematical tractability we choose the natural frequencies 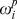 from an identical frequency distribution with mean 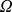 and standard deviation one, for every node 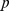.

### Measuring local and global synchrony

**Figure 3:**
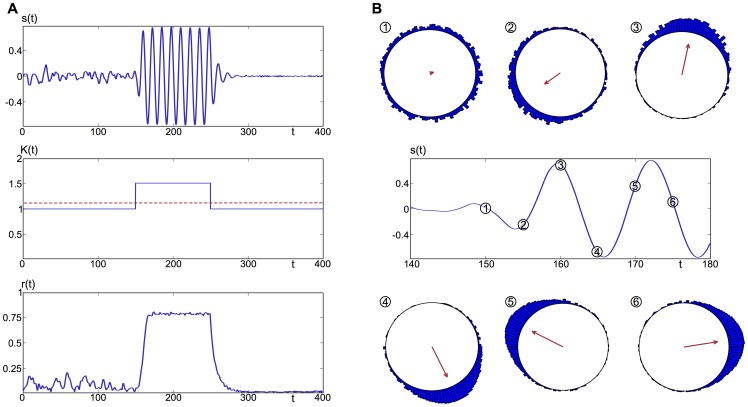
The Kuramoto model with varying coupling strength to demonstrate the ictal (synchronized) and interictal (unsynchronized) behavior of the model. **A**: For 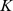 below a critical value 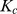 (red, dotted line) the signal 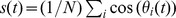 is irregular and the order parameter representing the degree of synchronicity is low. If 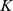 is above the critical value, 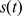 is sinusoidal with large amplitude, and the order parameter is large. **B**: At the onset of synchronization, the oscillators start forming a cluster resulting in an increase of the order parameter. Bars around the circles indicate the phase density of oscillators. The internal frequencies 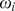 are drawn from a normal distribution with mean 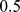 and standard deviation 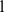. Here we use 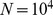 oscillators.

The degree of synchrony between oscillators within a single node and across the global network is controlled by the coupling parameters 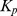 and 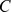. Focussing first on an individual node, Figure 3 demonstrates how the dynamic behavior of the Kuramoto model depends on the coupling constant 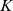. When this coupling constant is below a critical value, each oscillator behaves incoherently (i.e. they are uniformly spread around the unit circle) and the emergent signal is apparently stochastic and of low amplitude. However, when the coupling reaches a critical value, a phase transition occurs and oscillators become phase-locked (which in this context is synonymous to synchronized), resulting in emergent large amplitude oscillations; analogous to the transition between background and spike-wave activity seen in the onset of seizures.

To measure the degree of synchrony within the oscillators of an individual node 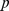, we use the order parameter 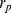 defined by:
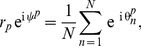
(3)which measures the level of phase coherence between all 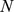 oscillators, where 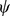 is the average phase. The order parameter is low (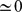) when oscillators are incoherent, and high (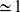) when they become coherent.

Using equation (3), we can reformulate equation (2) to obtain:
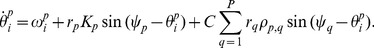
(4)Exploiting our assumption that the natural frequencies within each node come from the same distribution with mean 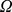, and that all connections in the network are either zero or positive, all ensemble averages will be in-phase (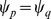 for all combinations 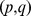) when 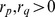, and consequently the following inequality holds:
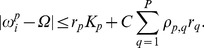
(5)Equivalently, we can find an expression for the phase difference between an individual oscillator and the ensemble average:
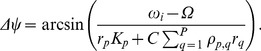
(6)In the thermodynamic limit (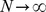) we can describe the distribution of natural frequencies with a function 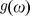. The order parameter is then the integral of the product of the density of natural frequencies and the cosine of the corresponding phase differences over all natural frequencies for which phase-locking occurs:
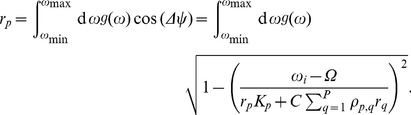
(7)The upper and lower limit of the integral in (7), 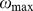 and 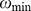, are determined from the inequality (5), resulting in:
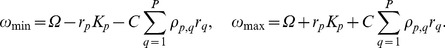
(8)By using the definition of Bessel functions we can evaluate the integral in (7), which yields an implicit equation for each node 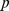:
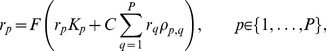
(9)where the function 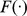 is given by:
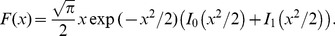
(10)


Having obtained an expression for the order parameter at the level of individual nodes, we now seek an expression for the order parameter across the network, which we term the *global order parameter*. The global order parameter is defined as
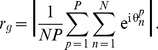
(11)This expression can be reformulated using (3) to obtain:
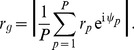
(12)Once more, all connections in the matrix 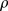 are non-negative, thus beyond the onset of synchronization the phases of the ensemble averages at each node are equal and we obtain
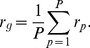
(13)Thus, the global order parameter is given by the average over 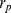, demonstrating that the degree of global synchrony can be inferred from the degree of local synchrony.

### Inferring functional networks from EEG

EEG recordings used in this study were collected from 35 people diagnosed with idiopathic generalized epilepsy (21 female, mean age 34.4 years), and from 40 controls (20 female, mean age 30.7 years) as previously described [56]. Ethical approval to use this data was obtained from Kings College Hospital Research Ethics Committee (08/H0808/157). Written informed consent was obtained from all participants. From these recordings, one artefact-free 20 second segment of background activity (eyes-closed (EC)) was extracted from each recording. The segments were bandpass filtered between *1*–*70 Hz*, and notch-filtered between *48*–*52 Hz* to exclude power line interference. The pre-processed data were then divided into frequency bands as given in Table 1. Whilst these frequency bands are different to those standard in a clinical setting (where the bands are defined according to prominent features visible to an expert observer), they are hypothesized to contain maximally-independent information representing different neurobiological generators [57]. Furthermore, given that brain network features in the alpha band may show evidence of heritability [58], [59], and that antiepileptic drug treatment my alter peak alpha frequency [60], this motivates the subdivision of alpha range following the work of [57].

**Table 1:** Frequency bands.

Frequency band	Range
delta	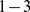 Hz
theta	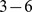 Hz
low alpha	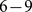 Hz
high alpha	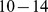 Hz
beta	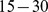 Hz
gamma	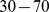 Hz

Frequency bands used in this study are motivated by the work of [57].

To infer the functional network structure from EEG recordings, we use a method based upon time-lagged cross-correlation [10] to infer weighted networks from the voltage signals of each electrode (see [61] for an evaluation of linear and non-linear methods for inferring functional connectivity). Our specific choice is motivated by the predominantly linear nature of resting-state EEG [62]. To account for false connections due to common sources, we only consider those connections with non-zero time-lag since previous studies have demonstrated that volume conduction primarily manifests as an instantaneous correlation [63], [64]. Entries for the connectivity matrix are given by
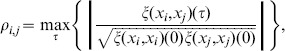
(14)with
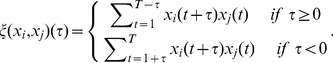
(15)


A potential source of spurious cross-correlation are autocorrelation effects due to finite length time-series data. To account for this, we create 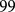 surrogate datasets from our original EEG data via the *iterative amplitude-adjusted Fourier transform* (IAAFT) method (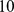 iterations) [65], which preserves autocorrelation whilst removing genuine pairwise cross-correlations within the time-series. Applying our method pairwise within each of these surrogate datasets creates a spectrum of cross-correlation values which could arise as a consequence of autocorrelation alone within the specific pair 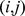. Therefore we reject connections 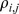 from the original EEG data if they do not exceed the 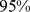 level of significance.

**Figure 4:**
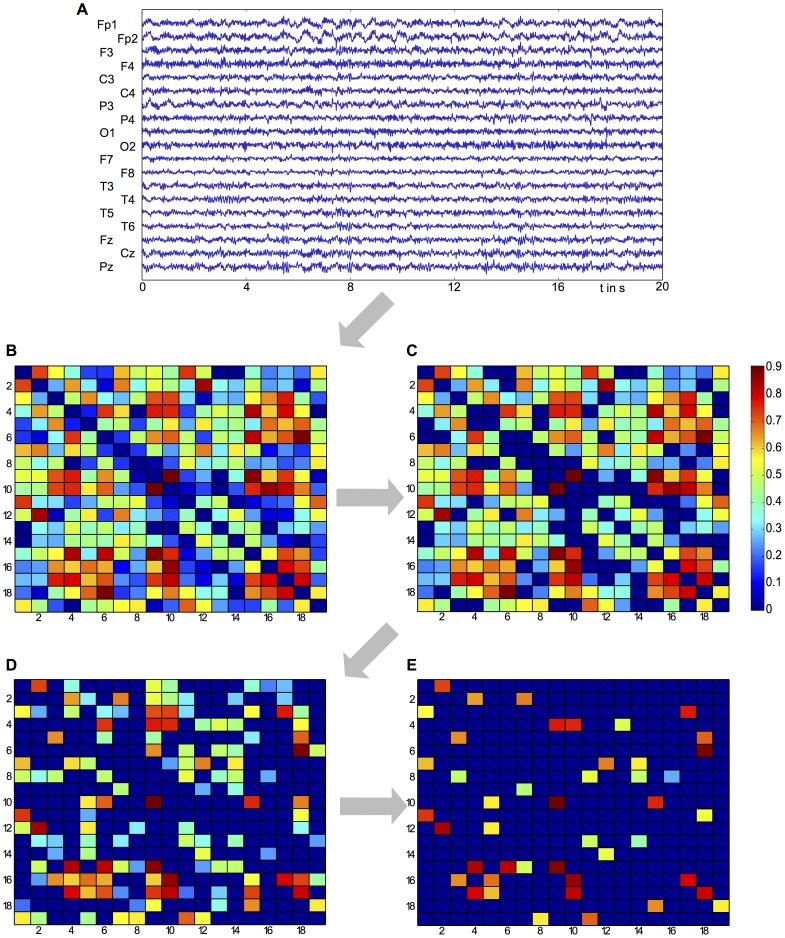
Illustration of the procedure to derive the functional network structure. **A**: An artefact-free 20s resting-state segment of EEG from each subject is extracted. **B**: Applying the time-lagged cross-correlation to all combinations of channel pairs yields a bidirectional connectivity matrix. **C**: Connections are removed if they are not significantly different from surrogate data (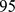
% level of significance). **D**: Using the time-lags, a unidirectional connectivity matrix can be inferred. **E**: Setting to zero all connections that can be explained by stronger, indirect connections removes spurious connections.

Next, we create a directional matrix by setting 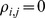 if 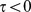, and 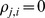 if 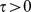. If 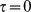, we set 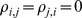 in order to remove zero time-lag connections. Further, we remove spurious connections by setting 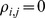 if, at first order, there exists a node 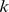 such that 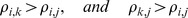. At second order, we set 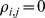 if there exist two nodes 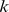,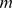 such that 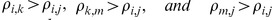. In other words, direct connections between nodes are removed if there exist stronger, indirect connections via one or two other nodes. A graphical representation of this procedure is given in Figure 4.

Finally, this functional connectivity matrix 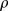 feeds into equation (2) in what may be thought of as an effective connectivity representation of the observed dynamics.

### Statistical analysis and the receiver operating characteristic

In order to test for statistically significant differences in the model-based measures at the group level, we use the Wilcoxon rank sum test [66]. In comparison to parametric tests, such as the t-test, this method does not assume the existence of an underlying normal distribution. The test yields the 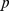-value (likelihood) that the medians of both samples are the same (null-hypothesis). As our analysis involves multiple hypotheses (frequency bands, nodes in the network) we correct the 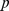-value using a conservative Bonferroni correction (effectively multiplying the 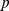-value by the number of hypotheses considered). If this corrected 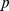-value is below 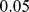, we consider the difference between the samples to be significant.

For model-based measures found to be significant on this basis, we then explore the discriminative power of the measure through computation of the receiver operating characteristic (ROC). The resulting ROC curve plots the true positive rate (TPR) against the false positive rate (FPR), which is achieved through varying the threshold (that parametrizes the ROC curve), and counting all sample points below this threshold as positives. Next, we identify the point on the curve which is closest to the the point of perfect classification, 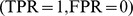, the upper left-hand corner. For this point, we compute the positive predictive value (PPV) defined as:
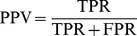
A PPV of 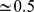 indicates the measure has no discriminative power, whilst a PPV of 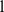 indicates perfect classification. The False Discovery Rate (FDR) is defined to be 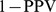.

## Results

### Conditions for the emergence of global synchrony

First, we address the conditions necessary for the global network of 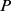 nodes, or a subset thereof, to synchronize. As for the case of the standard Kuramoto model, an individual node or a subset of nodes 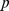 can synchronize if their intrinsic coupling strengths 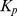 are greater than the critical coupling strength 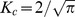. However, suppose that all nodes are individually in the desynchronized state (i.e. 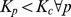). Does there exist a critical value 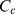 of the global coupling parameter 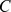 such that synchrony across some or all of the nodes in the global network emerges? To explore the existence of such a critical value 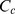, we first linearize (9) around 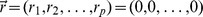 giving:
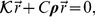
(16)where 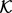 is a 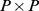-dimensional diagonal matrix with elements 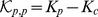, 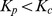. Trivially, the zero-solution for all order parameters (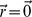) exists for any choice of 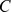. However, non-zero solutions for 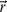 exist if the following determinant condition holds:
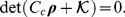
(17)Solving this determinant problem is computationally efficient in comparison to the corresponding full nonlinear problem (9). Alternatively, since 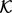 is invertible (as 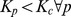), we can reformulate (16) as a standard eigenvalue problem with 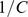 as eigenvalue:
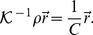
(18)As 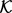 is diagonal, its inverse 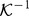 is diagonal as well, with elements 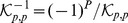. Finally, as all real-valued eigenvalues 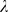 of 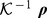 represent the inverse of a coupling constant 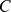 that permits non-trivial solutions around the zero-state, we identify the critical coupling constant with the inverse of the largest (real) eigenvalue 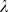 of 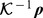:
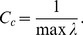
(19)We refer to this scenario as “network-driven synchronization”. Alternatively, if 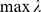 is zero, then no critical value of the global coupling exists, since 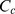 must be positive. This is the case when 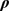 represents a network with hierarchical flow, and has upper triangular form. Here 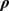 is nilpotent and all its eigenvalues are zero. In this scenario there may exist a node or nodes which, if synchronized (i.e. 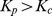), can drive other nodes (with intrinsic coupling parameters less than 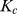) to synchronize due to the topology of the hierarchical network. We term this scenario “node-driven synchronization”. For the specific problem of epilepsy, we might consider these two scenarios equivalent to seizure onset as a distributed network property versus the existence of an epileptogenic zone, for example.

### Illustrative motifs

**Figure 5:**
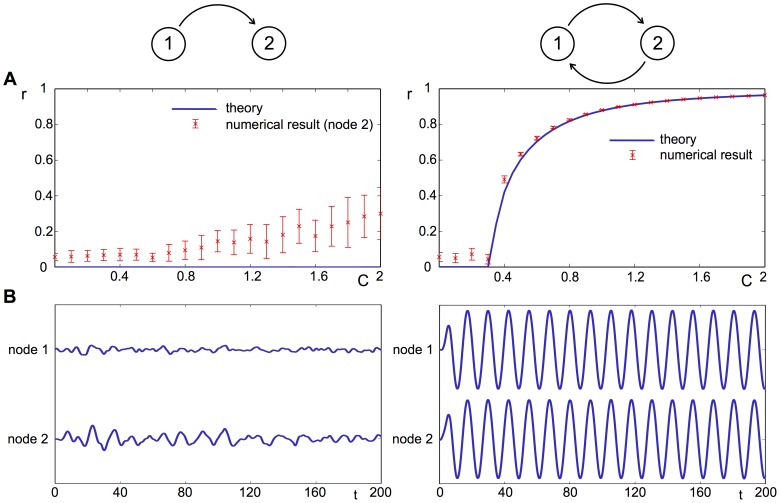
Model behavior on a two-node network. **A**: The order parameter 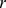 for unidirectional and bidirectional coupling between two nodes plotted against the global coupling parameter 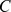, accompanied by numerical results. The slow increase of the numerical result of 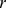 with 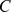 for unidirectional coupling is due to the finite size of the system with 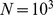 ocillators. **B**: The evolution of the signal 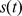 of each node with random initial conditions and 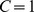.

To better understand these different conditions for emergent synchronization, we focus initially on motifs with a small number of nodes. First, we consider the case of two nodes that are either unidirectionally or bidirectionally coupled. In graph-theoretical terms, two bi-directionally coupled nodes are the simplest example of a feedback-loop or *cycle*, which, in turn, is the simplest form of a *strongly connected component*. A strongly connected component is a network configuration such that each node can be reached from all other nodes by following directed connections. Hence, a strongly connected component must contain at least one cycle. Conversely, two nodes that are uni-directionally coupled represent the simplest form of a network with a *hierarchical flow*, such that each node can be assigned its own level of hierarchy. First, we consider two coupled nodes in the most general scenario, where the connectivity matrix 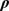 has entries 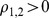 and 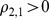. Then from (17) we obtain the following expression for the critical value of the global coupling parameter 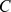, above which both nodes become phase-locked and their order parameters increase:
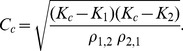
(20)Again, 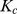 is the critical value of 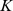 for the onset of synchrony within an individual node. 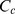 is only real-valued for 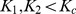, verifying that neither node is synchronized in isolation (a prerequisite for network-driven synchronization). In this scenario the value of 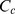 depends on the distance of the intrinsic coupling parameters from 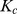. If both nodes are identical with 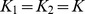, and 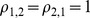 we obtain the simple expression
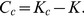
(21)U ni-directional coupling (i.e. either 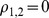 or 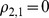) means (20) is undefined, making network-driven synchrony impossible. In this scenario, only node-driven synchrony can arise as a consequence of their intrinsic coupling exceeding the critical value 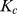. A comparison of both cases is shown in Figure 5, accompanied by numerical results for 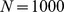 oscillators, which leads to an increasing divergence between analytical and numerical results for the uni-directional case with increasing global coupling.

We now generalize these ideas to larger networks, for which we make the distinction as to whether there exist cycles (or feedback loops, as per the bi-directional two node case) or a hierarchical structure (as per the uni-directional case).

#### Cycles and strongly connected components

**Figure 6:**
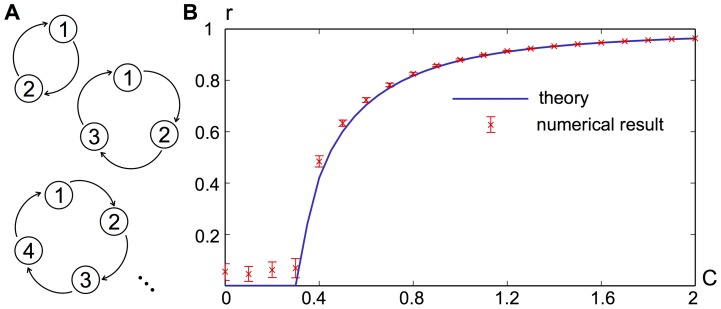
The order parameter on a cycle. **A**: Illustration of cycles with increasing number of nodes. **B**: A plot of analytical and numerical results of the order parameter on a cycle of 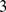 nodes. The numerical example is obtained for 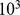 oscillators per node.

Strongly connected components are subsets of directed graphs in which there is a path from each node to every other node. Thus, each node in a strongly connected component has an in-degree greater than zero (i.e. it receives input from at least one other node), which is a critical property for enabling network-driven synchrony to emerge. Here, we focus on the example of a strongly connected component as a simple cycle (illustrated in Figure 6), with connectivity matrix 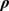 having elements 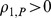 and 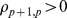 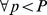, with all other elements zero. Once more, the intrinsic couplings 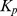 are chosen arbitrarily with the only restriction of 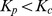. Using the linearization approach, we thus obtain
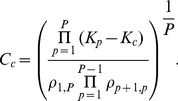
(22)As for the simple two node system, if all intrinsic couplings are equal (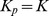), then the value of the critical coupling 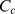 is the difference between 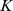 and 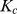. Likewise, if all intrinsic connections are identical, and all connections forming the cycle are equal to one, 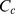 is simply defined by (21). Further, if any of the connections are removed and the cycle is broken, a zero is introduced into the denominator and 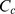 becomes infinite, making network-driven synchrony impossible. In real-world networks, a strongly connected component may be formed of more than one cycle, meaning it may be necessary to remove more than one connection to destroy it.

#### Networks with hierarchical flow

As for the simple case of two nodes, if a directed network has a hierarchical flow, then the corresponding connectivity matrix has upper triangular form and its determinant is zero. Consequently, there exists no critical value 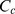, and thus network-driven synchronization is impossible. In this scenario, only node-driven synchrony can occur.

Given that for increasing node size the number of possible network combinations rapidly becomes very large, we present an example that illustrate s how subtle changes in the structure of directed networks can create or destroy strongly connected components, which in turn lead to the emergence of synchrony where previously there was none, or vice versa.

**Figure 7:**
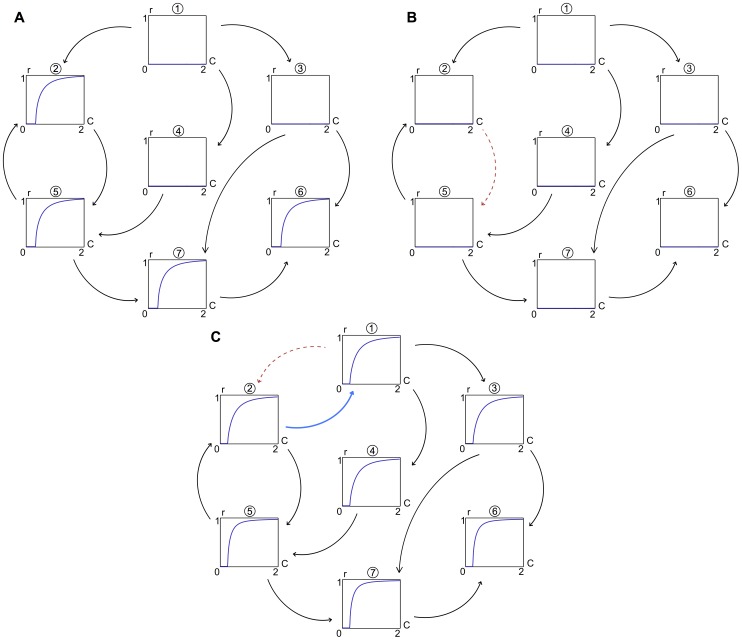
Illustration of how subtle changes in the network structure affect the ability of the network to synchronize. **A**: An arbitrarily chosen network shows partial synchronization due to a cycle (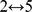) and two adjacent nodes (6,7). **B**: By removing one connection (red, dashed) the cycle is broken and the network loses its capability for synchronization. **C**: By reversing the connection between 1 and 2 (blue, bold), the network from **A** becomes globally synchronous for large enough 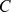. Numerical results are in agreement with analytical results, but omitted here. The intrinsic coupling constant of all nodes is set to 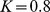.

In Figure 7**A** we present a binary network of seven nodes, in which a sub-network synchronizes for 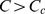. This emergent synchrony occurs through a combination of a cycle and the network structure that connects nodes within the cycle to other nodes. Nodes that do not receive input from the cycle, either directly or indirectly, remain unsynchronized. By removing one connection (Figure 7**B**) we break the cycle and the emergent synchrony is lost, as it has become a purely hierarchical network. On the other hand, through changing the directionality of another connection (Figure 7**C**), synchrony emerges across all nodes (not just the sub-network) for 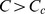 as a consequence of the existence of a strongly connected component (involving nodes 1, 2, 3 and 5), which connects to all other nodes.

### Network structure and epilepsy

Having studied the conditions necessary for the emergence of global synchrony to be network or node-driven, we now apply this understanding to functional networks inferred from EEG recordings collected from our cohorts of people with epilepsy and controls. For each individual and each frequency band we obtain a functional connectivity matrix 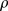, where each node within the network corresponds to a specific EEG channel. We study these matrices from two perspectives: First, we consider the critical value for the global coupling parameter 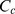, above which network-driven synchrony emerges and compare these values for functional networks inferred from people with epilepsy and those inferred from controls. Where the critical coupling strength required to enable global synchrony is smaller, this suggests that those networks are more seizure prone than others. Second, we study whether there exist specific nodes within these functional networks which may drive emergent synchrony across the wider network. This latter study is motivated by recent studies from human and rodent models that suggest generalized seizures in IGE appear to have a focal onset [67]. Here, we set each node to be self-synchronized, and analyze the effect this has on the rest of the network by computing the global order parameter, which indicates the global degree of synchronicity.

#### Network-driven synchrony

**Figure 8:**
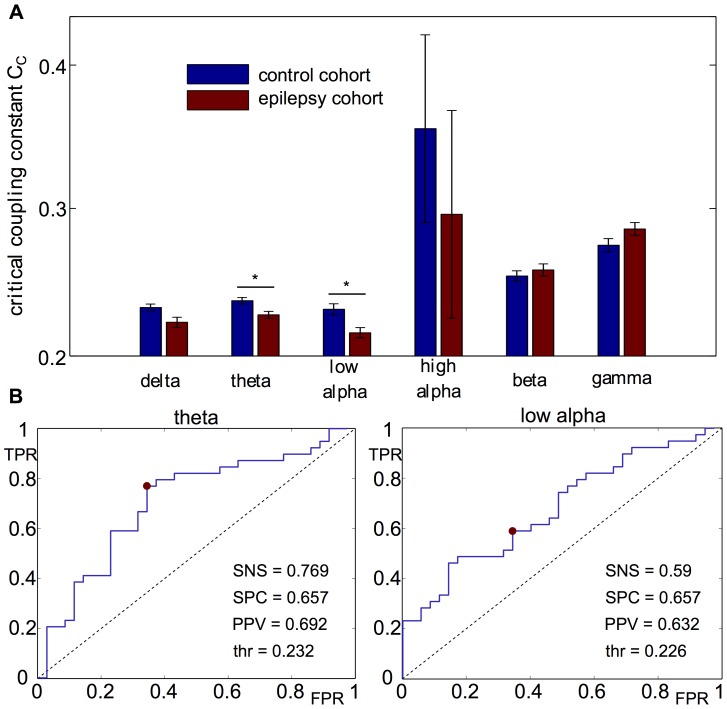
Critical coupling constants in the functional networks obtained from the epilepsy cohort and the control cohort in different frequency bands. **A**: A significantly lower 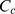 in the theta and low alpha band indicates that the functional network in the interictal state of the epilepsy cohort is closer to synchronization than in the control cohort. Interestingly, ictal discharges occur in the theta band as well. Level of significance: 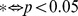. Error bars indicate the standard error of the mean. 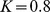. **B**: Receiver operating characteristic for the detection of members of the epilepsy cohort through use of thresholded values of 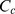 as the discriminating factor for networks inferred from either the theta or low-alpha band. The red dot indicates the point with best discrimination, which is the point closest to the point of perfect classification (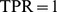,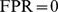). Abbreviations: FPR - false positive rate, TPR - true positive rate, SNS - sensitivity, SPC - specificity, PPV - positive predictive value, AUC - area under the curve thr - threshold for discrimination.

For each frequency band and each cohort (epilepsy and controls), we determine a set of critical values 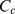 for the emergence of network-driven synchrony. For our simulations, we fix all intrinsic coupling constants, 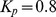, which is less than the critical value for self-synchronization, 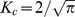. Using the Wilcoxon rank sum test, we find a statistically significant reduction in the mean value of the critical global coupling parameter 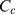 for functional networks from the epilepsy cohort in both the theta (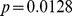) and low-alpha band (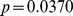). This implies that the functional networks of people with epilepsy drive global synchrony more readily than those from controls. Since, at the macroscale, epilepsy is associated with the emergence of hypersynchrony across large-scale brain regions, this demonstrates a possible mechanism by which seizures can emerge in people with epilepsy as a consequence of brain network structure. The mean values for each set are shown in Figure 8, along with an annotation of the level of statistical significance of the difference. Moving from these group level analyses, we then examined the potential for individual discrimination using receiver operating characteristic (ROC) analysis. In this case ROC analysis shows some predictability at the individual level with a positive predictive value (PPV) of 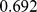 in the theta band, and a PPV of 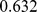 in the low alpha band. This corresponds to a false discovery rate (FDR) of 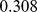 and 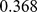 respectively. Values for sensitivity and specificity are presented in Figure 8.

#### Node-driven synchrony

Motivated by experimental evidence that generalized spike-wave discharges can emerge from a focal onset zone [67], we next investigate whether there exist particular nodes within the inferred functional networks, which, when synchronized, can drive higher levels of synchrony across the global network in patients compared with controls. To explore this, we systematically set the intrinsic coupling strength 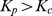 for each node 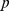, such that node 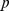 synchronizes, and study the effect of this on global synchrony across the whole network measured through an average over all local order parameters, 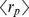, which for our model setup is equivalent to the global order parameter (13).

**Figure 9:**
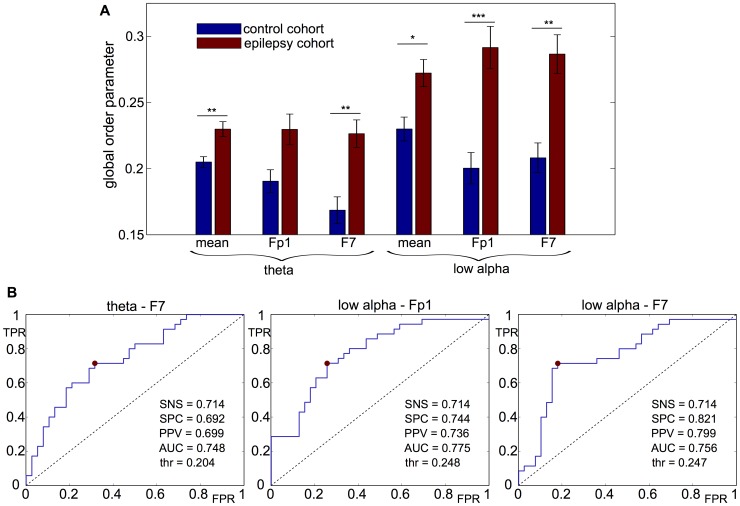
The global (average) order parameter of the network when one node is self-synchronized. **A**: We show here the result for self-synchronization in Fp1 and F7, and also the average over all electrodes, in the theta band and low alpha band. Other electrodes are omitted as they do not yield significant results when p-values are Bonferroni-corrected by a factor of 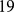 (the number of electrodes). This finding confirms the result of previous studies (see text) that identified frontal and pre-frontal areas as seizure onset zones. Levels of significance: 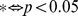; 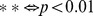; 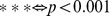. Parameters: 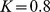 for all nodes except self-synchronized node with 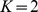; 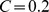. Error bars indicate the standard error of the mean. **B**: Receiver operating characteristic for the detection of the epilepsy cohort by using the global (average) order parameter as discriminating factor in F7 in the theta-band, and Fp1 and F7 in the low-alpha band. Again, the red dot indicates the point with best discrimination. Abbreviations as per Figure 8.

We find that averaging 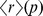 over all nodes 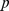 yields significantly larger values for people with epilepsy than for controls, in both the theta band and the low - alpha band. This is analogous to the network-driven scenario; demonstrating that global synchrony within networks of people with epilepsy is more easily driven by hyperexcitability within specific nodes in comparison to controls. At the level of individual nodes, after Bonferroni correcting for the number of individual nodes varied (19), we find that the node corresponding to electrode F7 in the theta band, and the nodes corresponding to electrodes Fp1 and F7 in the low alpha band have a significantly stronger synchronizing effect on the global network in people with epilepsy compared to controls (again using the Wilcoxon test), see Figure 9. The p-values are 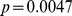, 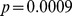, and 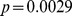 respectively. This finding that frontal areas may initiate seizures is consistent with several previous studies [53], [68]–[76], using different imaging modalities.

Once more we also explore the ability of the model to discriminate at the individual level. Here the ROC analysis shows greater levels of predictability than using the global coupling parameter and network-driven synchrony as a discriminator. In particular, using F7 shows a very promising PPV in the low-alpha band of 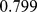. Full details of the ROC analysis are presented in Figure 9.

## Discussion

We have previously described a phenomenological approach to studying network abnormalities in epilepsy from a purely theoretical standpoint [42], illustrating that seizures could occur due to either abnormalities in the dynamics of brain regions or the connectivity structures between them. Here, we advance this understanding using a modular network of embedded Kuramoto oscillators, that has enabled us to explore the interplay between node dynamics and network structure in the emergence of hypersynchrony, analogous to the generation of seizures, both in theory and in real data. We have derived necessary conditions for the emergence of synchronization within large scale networks in terms of the pattern of directed edges in the network, the intrinsic coupling parameters within each node, and the macroscopic coupling parameters between nodes. Specifically, we demonstrate that strongly connected components (i.e. disparate regions that form complete cycles) are necessary for the emergence of global synchrony for a collection of nodes that individually are sub threshold, whereas directed networks with hierarchical flowcan only result in global synchronization if nodes at the top of the hierarchy become synchronized themselves. In binary networks, strongly connected components can be created by adding connections to an existing network, or they can be destroyed by removing specific connections. In general, an indicator of the degree of network-driven synchronization is the critical value of the global coupling parameter 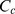; a small 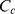 indicates a strong disposition for nodes to synchronize due to the particular structure of the network.

In larger, weighted networks we might reasonably expect to observe mixtures of strongly connected components and hierarchical subnetworks. Indeed, this is demonstrated in our results of functional networks inferred from EEG data. On the one hand, we find that the critical coupling strength, that is necessary to enable the emergence of synchrony in the global network, is significantly lower for networks inferred from the EEG recordings of our cohort of people with epilepsy in comparison to healthy controls. This indicates an increased presence of strongly connected components in the network and suggests a fundamental mechanism for the tendency to experience recurrent seizures in people with epilepsy. On the other hand, we observe that left frontal brain regions (represented by EEG channels Fp1 and F7) can drive increased levels of global synchronization when they are self-synchronized, which indicates an increased presence of hierarchical flows as well.

These latter findings, that generalized seizures may be driven by activity in left frontal regions of the brain, complement previous findings using other imaging modalities, for example Pavone and Niedermeyer [71] who identified a cortical, mostly frontal lobe involvement in absence seizures and primary generalized seizures. Likewise, Holmes *et al.* [77] and Amor *et al.* [72] identified frontal areas as highly involved during absence seizures. This evidence is supported by the fact that working memory - a frontal lobe function - is suspended during typical absence seizures. A critical advantage of our approach is that these differences are identified from epochs of data from inter-ictal time-periods (i.e. away from seizures).

MRI studies [73]–[76] in one particular IGE syndrome, juvenile myoclonic epilepsy, have identified a structural abnormality of medial frontal cortex, and abnormalities of structural connections (using DTI) and functional connections (using fMRI) of this area with motor cortex, frontopolar cortex, thalamus and contralateral medial frontal cortex, supporting the EEG/MEG data implicating frontal abnormalities. Further, Yan and Li [53] inferred human brain networks from diffusion-magnetic resonance imaging in healthy controls, and postulated that frontal hubs could drive seizure activity when placing these data inferred networks onto a computational model utilizing a delayed version of the Kuramoto model. Our study extends this research by comparing the networks of people with epilepsy directly with those of healthy controls, and demonstrating an increased propensity for seizure generation as a consequence of the functional network structure. This current study complements our earlier work [41], [42], where we used an alternative model formulation (a subcritical Hopf bifurcation to reflect the rapid transition from background activity to seizures) to explore the role of network structure in driving the onset of seizures.

Our present study has focussed on analysis of routine clinical EEG in ‘sensor’ space, by which we mean functional networks were inferred through studying the interactions spanning EEG electrodes, rather than the interactions between the brain sources responsible for generating the activity. This is necessitated by the limited spatial sampling of the clinical data (19 channels) that does not readily permit the use of source reconstruction techniques [78]. Volume conduction can be a further concern when utilizing data from scalp electrode recordings. To compensate for this, we used a time-lagged cross correlation function (excluding the zero time lag) for inferring functional networks. It is therefore important that future work should extend our approach to larger networks inferred from either high density EEG, fMRI or DTI data. Given that we determine the point of onset of global synchronization using an analytic expression, our framework can be applied naturally to networks of any size. Further research should also seek to understand long-term disease progression through studying longitudinal clinical recordings collected at regular intervals post-diagnosis, or in response to changes in treatment. Here, alterations in network structure or dynamics may point towards remission or successful drug response.

Of further importance is the non-trivial relationship between structural, functional and effective connectivity. For example, a large repertoire of functional networks can be supported by the same underlying structural network [79]. Further, effective connectivity is dependent both on the choice of generative model, as well as the observation data. Thus, effective connectivity should not be thought of as a unique representation of our data, and more likely there can be different (model, network) pairs that are consistent with the observed functional connectivity structure. As a simple example of this, consider the auxiliary approach for detecting generalized synchronization introduced by Abarbanel et al [80]. Here, a system 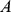 drives a system 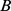, and the dynamics of these systems may become coherent depending on the nature of the coupling. If the dynamics of 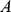 and 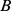 are chaotic, then this coherent relationship is highly non-trivial. However, by considering a copy of system 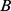, Abarbanel and colleagues show that one can infer the existence of this synchronization between 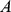 and 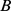, by observing that it is possible to infer a much simpler functional relationship between 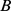 and its copy 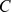, even though there is in reality no direct connection between them. Now reverse-engineering this scenario, suppose that we can only observe 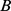 and 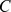, with no knowledge of system 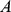. As an effective connectivity structure, we would identify a bidirectional link between 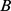 and 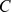 with a model representation of the simple functional form. However, in reality, there is an alternative effective connectivity structure that links 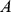 to 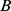 and 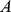 to 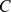 with the original more complex relationship. Whilst it has been shown that there can still exist a wide repertoire of functional networks [81], we might reasonably expect differences across cohorts to become apparent in resting-state functional networks at the group level. The inherent variability in functional expression may reflect the overlap between the patient cohort and the control cohort.

In conclusion, our findings are significant for a number of reasons. First, they demonstrate the power of pursuing a computational modeling approach to elucidate the mechanisms underlying differences observed in graph theory measures of data inferred functional brain networks. In this regard, the approaches we describe may have potential for understanding inferred brain networks from other neurological conditions, for example dementia (for which there is a strong association with seizures [82]) and schizophrenia [83]. Second, our study was performed inferring functional networks using epochs of background activity (i.e. away from seizures), which suggests that network structure is an enduring and critical marker of the propensity for seizures that offers the potential for diagnosis of epilepsy without the need to induce seizures within the clinical environment. Indeed, in this regard, beyond the group level differences we identify, the ROC analysis we performed demonstrated up to 80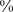 predictive power of this method for discriminating at an individual level, despite neither the model nor data being optimized for this purpose. This strongly motivates the potential of this approach. Third, our methods identified these networks using routine clinical EEG recordings, with low spatial and temporal sampling. Despite these apparent limitations, our study identified candidate regions that drive the onset of seizure activity, which are consistent with those obtained using more expensive MEG and fMRI modalities. Finally, deriving a mathematical equation for the global synchrony of the network makes it computationally tractable to analyze patient data in close to real time (through removing the need to numerically simulate large networks of oscillators). Taken collectively, these findings suggest that a computational modeling approach to analyze routine clinical data can be used in real time within the clinic as a diagnostic aid for clinicians treating epilepsy, as well as other neurological disorders, for which synchrony may potentially play a role.
